# Severe Rhabdomyolysis in a Pediatric Patient after Coxsackie B Virus Infection without Acute Renal Failure: A Case Report

**DOI:** 10.7759/cureus.7126

**Published:** 2020-02-28

**Authors:** Ahmad Soliman, Supriya Bisht, Kokila Jeyamurugan, Palanikumar Balasundaram, Ratna Basak

**Affiliations:** 1 Pediatrics, Brookdale University Medical Center, New York City, USA

**Keywords:** rhabdomyolysis, creatinine phosphokinase, coxsackie

## Abstract

Rhabdomyolysis is a condition resulting from the breakdown of skeletal muscle fibers with leakage of muscle enzymes into the circulation. The degraded muscle components in the circulation can lead to lethal complications as acute renal failure (ARF). In younger children, viral infections tend to be the major cause while trauma and exercise are the important ones in adolescents. Several viruses such as influenza A & B, parainfluenza and coxsackie have been implicated in causing rhabdomyolysis.

We report a case of a 14-year-old girl with severe rhabdomyolysis after recent Coxsackie B infection without acute renal failure.

## Introduction

Rhabdomyolysis is a potentially life-threatening syndrome characterized by striated muscle dissolution or disintegration into the blood stream [1]. It can develop from a wide variety of causes. Viral infections are the major causes in younger children while trauma and exercise are the main etiologies in older ones. Many viruses have been reported to cause rhabdomyolysis such as influenza A/B, parainfluenza, coxsackie, Epstein-Barr (EBV), herpes simplex, adenovirus, and cytomegalovirus [2].

We report a case of a 14-year-old girl with severe rhabdomyolysis after Coxsackie B viral infection. Many studies correlate elevated levels of creatinine phosphokinase (CPK) to acute renal failure (ARF) [3-7]. However, our patient did not show any signs of ARF or deranged renal function tests despite having very high levels of serum CPK.

## Case presentation

A previously healthy 14-year-old girl presented to the emergency department with history of passing red-colored urine for two days. She had coryza and body ache for four days. She denied abdominal pain, dysuria, recent trauma, sore throat, heavy exercise, fever, rash or any substance or drug abuse. She had taken one dose of ibuprofen 200 milligram for body ache.

**Figure 1:**
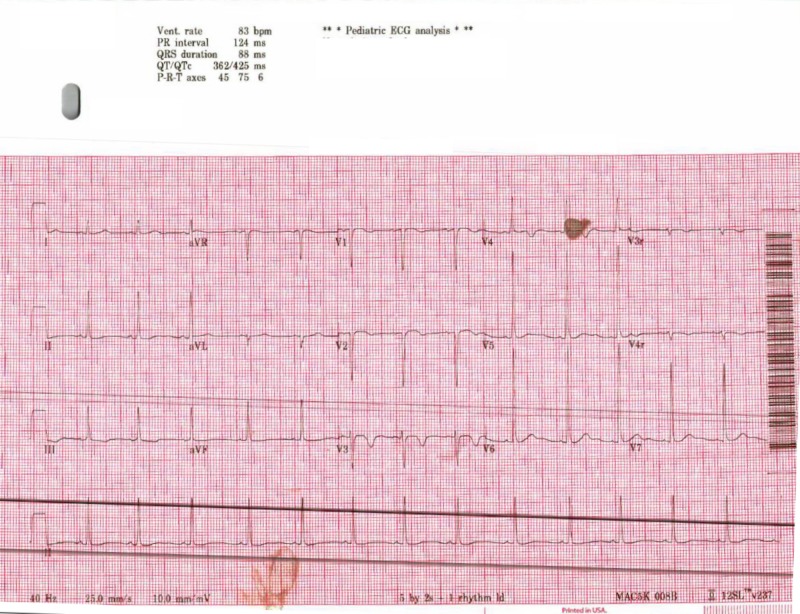
Electrocardiogram showed nonspecific ST and T wave changes

On admission, her vital signs were stable. Physical examination was unremarkable except for mild tenderness over her shoulders. There was no swelling or tenderness in her lower extremities which had full range of motion. Urinalysis (UA) showed large blood and 3-5 red blood cells per high power field. CPK was reported as more than 160,000 U/L. Alanine transaminase (ALT) and aspartate transaminase (AST) were 327 U/L and 1363 U/L, respectively. Complete blood count, renal function tests, serum electrolytes (including potassium, calcium, and phosphorus), uric acid, blood gas, coagulation profile, and urine electrolytes were within normal limits. Drug abuse urine screen was negative. Acetaminophen and salicylate levels were normal. Hepatitis serology and Group A Streptococcus screen were negative. Electrocardiogram showed nonspecific ST and T wave changes (Figure 1) but echocardiogram was reported normal. EBV, Influenza A/B, Coxsackie A/B, and HIV serology were sent.

**Figure 2:**
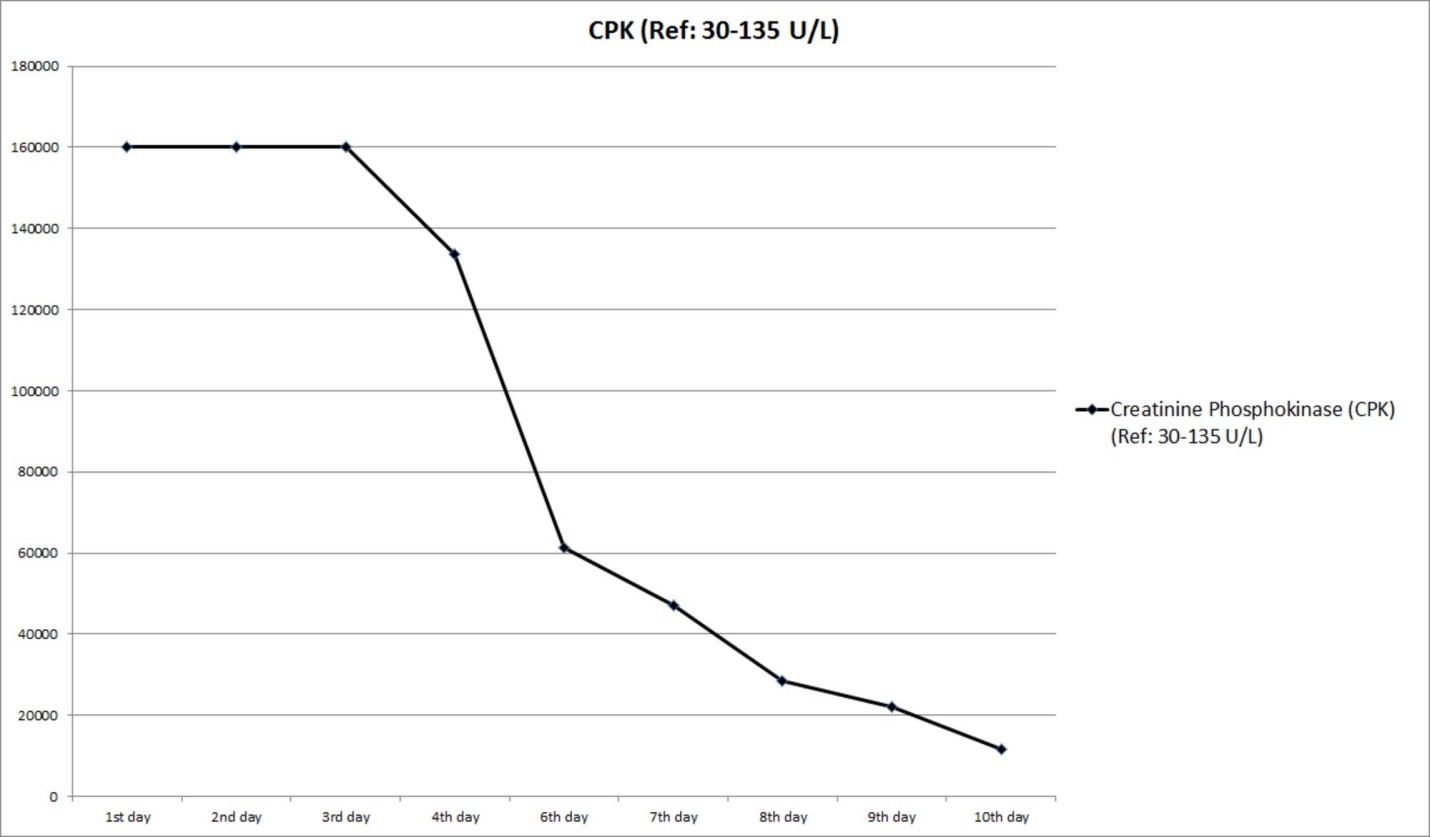
Trend of creatinine phosphokinase (CPK) values during the hospital stay

She was aggressively hydrated with two normal saline boluses in the emergency department. She was continued on two times maintenance fluids with normal saline in the inpatient floor. Bicarbonate infusion was not commenced as pH was 7.46 and bicarbonate level was 31.0 mmol/L in the venous blood gases. She was kept on strict input-output instructions, bed rest and was monitored for fluid overload. Repeat CPK was still more than 160,000 U/L on the second and third day of the hospital stay. They showed a declining trend on the fourth day and thereafter (Table 1, Figure 2).

**Table 1:** Laboratory values of creatinine phosphokinase (CPK), blood urea nitrogen (BUN), serum creatinine, serum alanine transaminase (ALT), serum aspartate aminotransferase (AST) during the hospital stay

	Creatinine Phosphokinase (CPK) (Ref:30-135 U/L)	Blood Urea Nitrogen (BUN) (Ref: 0.5-17 mg/dL)	Serum Creatinine (Ref: 0.52-1.04 mg/dL)	Serum Alanine Transaminase (ALT) (Ref: 9-52 U/L)	Serum Aspartate Aminotransferase (AST) (Ref: 14-36 U/L)
1st day	>160,000	11	0.84	327	1363
2nd day	>160,000	3	0.47	368	1408
3rd day	>160,000	4	0.45	532	1408
4th day	133,626	4	0.44	510	864
6th day	61,382				
7th day	47,012	5	0.38	390	389
8th day	28,591				
9th day	22,165				
10th day	11,674	4	0.4		

**Figure 3:**
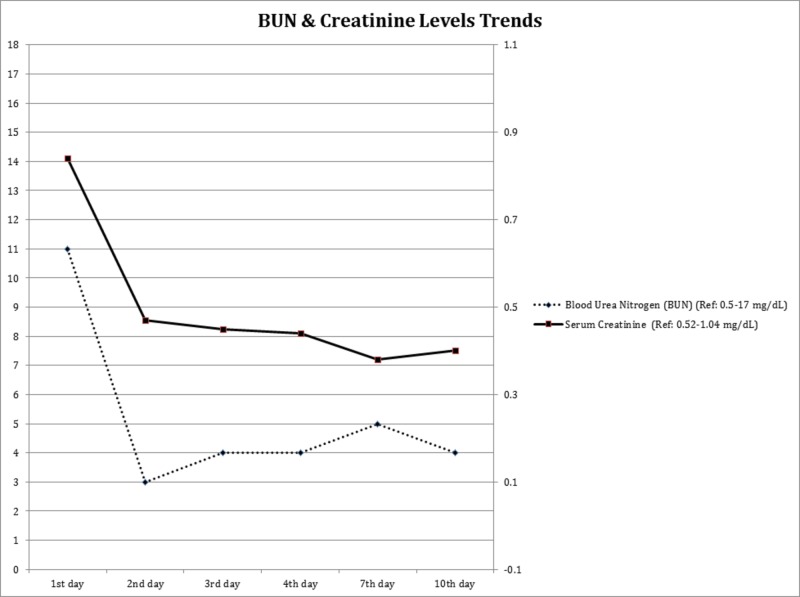
Trends of renal function tests during the hospital stay

ALT and AST levels trended down (Table 1). Renal function tests remained normal throughout (Figure 3).

Serum ammonia, lactate, pyruvate and plasma acylcarnitine, which were drawn to rule out the other etiologies, were negative. Coxsackie B antibodies were positive (B1, B5, and B6 were 1: 64, while B2, B3, and B4 were 1: 32 positive), while the Coxsackie A antibodies were negative.

On the tenth day, CPK trended down to 11674 U/l, parenteral fluids were weaned down and oral fluid intake was encouraged. She was discharged on the following day. One week later, she was followed up in the outpatient department and her blood test results were back to normal.

## Discussion

Rhabdomyolysis is a syndrome of muscle necrosis and release of muscle components in the circulation and urine. Most of the literature on rhabdomyolysis is drawn from adult studies. The data on pediatric rhabdomyolysis is very limited [2].

The most common causes of rhabdomyolysis in adults are muscular trauma and exogenous toxins [7,8]. Trauma is the most common etiology in older children [2]. In young children under nine years, viral infection is the most common cause [2]. Viruses may cause rhabdomyolysis by directly attacking the muscle and generating muscle-specific toxin. Influenza A&B and enteroviruses (Coxsackie A&B) were found to be the most common etiology [2,9,10]. Other viral agents that were reported to cause viral myositis or rhabdomyolysis include Epstein-Barr virus (EBV), human immunodeficiency virus (HIV), and herpes simplex virus [11-13].

Coxsackie B is a group of six serotypes of coxsackievirus which are under enteroviruses. They can present from being asymptomatic to causing severe myalgia and muscle pain. Initial symptoms may be non-specific symptoms such as fever, malaise, headache, and gastrointestinal upset. They may also cause severe chest and muscle pain known as pleurodynia. They account for 50-90% of aseptic meningitis cases.

Our patient had mild non-specific symptoms like malaise, headache, and rhinorrhea for two days before passing red-colored urine with very high serum CPK levels. Although many studies correlate elevated levels of CPK to ARF, our patient did not show any signs of ARF or deranged renal function tests [3-7]. This is the first instance reported in the literature where such high CPK levels in a pediatric patient with Coxsackie B rhabdomyolysis, did not lead to ARF.

## Conclusions

Viral-induced myositis appears to be the most common etiology for rhabdomyolysis in younger children. Among viral infections, influenza A&B and enteroviruses (Coxsackie A&B) were found to be the most common etiologies. Absence of ARF in a pediatric patient with Coxsackie B rhabdomyolysis and such high CPK levels, is not reported in the literature previously. Testing for viruses including Coxsackie A or B should be considered while investigating a child with severe non-traumatic rhabdomyolysis.
